# Future Treatment of Neuropathic Pain in Spinal Cord Injury: The Challenges of Nanomedicine, Supplements or Opportunities?

**DOI:** 10.3390/biomedicines10061373

**Published:** 2022-06-10

**Authors:** Giuseppe Forte, Valentina Giuffrida, Angelica Scuderi, Mariella Pazzaglia

**Affiliations:** 1 Dipartimento Di Psicologia, Sapienza Università Di Rome, 00185 Rome, Italy; angelica.scuderi@uniroma1.it; 2 Body and Action Lab, Istituto di Ricovero e Cura a Carattere Scientifico Fondazione Santa Lucia, 00179 Rome, Italy; valentina.giuffrida@uniroma1.it

**Keywords:** pain, neuropathic pain, nanomedicine, spinal cord injury, nanotechnology

## Abstract

Neuropathic pain (NP) is a common chronic condition that severely affects patients with spinal cord injuries (SCI). It impairs the overall quality of life and is considered difficult to treat. Currently, clinical management of NP is often limited to drug therapy, primarily with opioid analgesics that have limited therapeutic efficacy. The persistence and intractability of NP following SCI and the potential health risks associated with opioids necessitate improved treatment approaches. Nanomedicine has gained increasing attention in recent years for its potential to improve therapeutic efficacy while minimizing toxicity by providing sensitive and targeted treatments that overcome the limitations of conventional pain medications. The current perspective begins with a brief discussion of the pathophysiological mechanisms underlying NP and the current pain treatment for SCI. We discuss the most frequently used nanomaterials in pain diagnosis and treatment as well as recent and ongoing efforts to effectively treat pain by proactively mediating pain signals following SCI. Although nanomedicine is a rapidly growing field, its application to NP in SCI is still limited. Therefore, additional work is required to improve the current treatment of NP following SCI.

## 1. Introduction 

**Figure 1:**
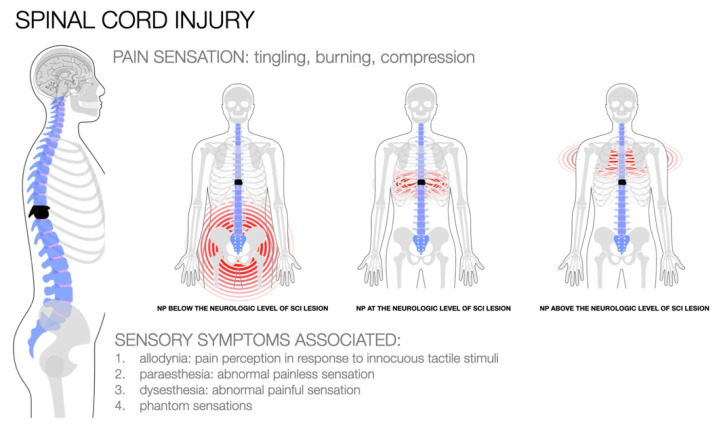
Clinical aspects of neuropathic pain following spinal cord injury. NP within the dermatome of the neurological injury and up to three levels below the neurological injury level, diffusely more than three dermatomes below the level of the neurological lesion.

Neuropathic pain (NP) is a common, severe, disabling chronic condition that affects people and is notoriously difficult to treat [1,2]. This pain typically occurs as a result of a lesion or inflammation of the nervous system, potentially affecting the peripheral nerve, dorsal root ganglion or root, or central nervous system [1,2,3,4]. NP may be caused by abnormal heterotopic activity of the injured nerve, peripheral and central sensitization, excitatory-inhibitory regulation imbalances, and microglial activation [5,6,7,8]. Additionally, NP is frequently characterized by persistent pain that is not triggered by external stimuli, frequently described as a lancinating burning sensation associated with increased and decreased sensory signs [9], most commonly, aberrant sensations known as allodynia [10] (pain perception in response to innocuous tactile stimuli), paraesthesia (abnormal painless sensation), dysesthesia (abnormal painful sensation) [5,6,7,10], and phantom sensation [11]. Given its heterogeneity, NP effectively represents a difficult pain category to treat and categorize [12,13]; therefore, the best diagnostic approach is to interview the patient, identify the underlying causes, and make exclusions. Treatment is individualized for each patient to optimize pain control and improve their functional status [14]. Although more diseases (e.g., diabetes, immunodeficiency, malignancies, traumatic and ischemic disorders) are associated with NP, individuals with spinal cord injuries (SCI) may be even more vulnerable [5,6,7,10]. SCI is a catastrophic neurological injury that frequently occurs as a result of sudden spine trauma and results in fractures and vertebral dislocations [3,5], affecting the continuity and conduction of the central nervous system, leading to a complex pathophysiology. Clinical sequelae depend on the severity and level of the spinal lesion. Primary injury occurs immediately following the injury and involves the destruction of neural parenchyma, axonal network, and glial membrane as a result of spinal cord compression [5,6,7,8]. The onset of biochemical, mechanical, and physiological changes within neural tissues initiates a sustained cascade of biological events referred to as a “secondary injury” [15]. A disrupted signal propagates from the lesion’s origin in the spinal cord to the sensorimotor tract’s termination in the cortex [7,8,9], resulting in changes at all levels of somatosensory circuitry (i.e., the spinal cord, brainstem, and brain). These dynamic processes in the central circuitry alter and modify the sensorial communication between the brain and the body [11,16,17], resulting in the development of various degrees of sensory and motor impairment and aberrant pain sensations above, below, or at the level of the lesion [16,17] (Figure 1). 

SCI is a frequent condition that affects nearly 20 million people worldwide, increasing by approximately 700,000/900,000 patients per annum [8]. The treatment must be started as soon as possible to treat the medical, physiologic, and psychological consequences of the injury and is costly and exhausting, posing a huge burden on patients, families, and healthcare systems. In this context, it is important to research better therapeutic alternatives geared toward reducing treatment duration and improving the functional state and emotional comfort that impact community participation and quality of life [14].

Around one-third of people with SCI experience persistent and severe pain, with NP being the most prevalent type, occurring in up to 96% of patients [18,19]. NP typically manifests within the first year following SCI [20], is resistant to nonpharmacologic interventions such as surgery, neurostimulation, and physical and psychological therapy [21,22,23,24], and is associated with increased drug prescriptions and health care provider visits [25]. Since NP has a negative impact on a patient’s daily activities, quality of life, mood, and rehabilitation outcome, the Food and Drug Administration (FDA) has approved a variety of drugs and pharmacological treatments for NP [26,27,28]. Thus, pharmacologic interventions, such as antidepressants, anticonvulsants, and psychotropic medications, continue to be the cornerstone of SCI pain management [29,30]. However, none of these interventions have long-term benefits, and NP is less responsive to opioids and other analgesics [31], implying that current pharmacologic treatment is frequently insufficient. As a result, there can be no resolution at the moment, resulting in a pain reduction of only 20–30% in intensity and only in one-third of patients [30].

Nanomedicine may be able to address potentially dangerous side effects, allowing for more precise and targeted treatments without the drawbacks associated with current clinical pain therapies [32]. Compared to conventional treatment, nanodrug delivery systems with proper design have demonstrated numerous advantages, including drug transport across the blood–brain barrier (BBB); increased solubility, stability, and half-life; controlled release of drugs; selective targeting; and protection of drugs from immune degradation [33]. However, the majority of published research on nanoparticle-mediated targeting has focused on cancer, and there has been a delay in applying nanoscience to pain management [34,35,36,37]. It is hypothesized that the concept of active and passive targeting of nanoparticles applies to all types of focal pathologies, including NP, which has been shown to alter BBB permeability via activation of microglia and astrocytes, as well as overexpression of glial receptors [38,39,40], thereby impairing therapy effectiveness. Given the complexity of pain physiology in SCI and the intractable nature of chronic NP, nanodrug delivery has the potential to play a significant role in the next generation of NP treatment, particularly following SCI. Several nanoformulations have entered clinical trials in recent years, and a few have been approved for commercialization by FDA [41]. While numerous reviews have been published in the field of nanomaterials for drug delivery, few have discussed the efficacy of nanomaterials in the management of NP, particularly in SCI [41,42,43,44]. Thus, this perspective review discusses novel treatment approaches targeting brain mechanisms to alleviate pain due to SCI and highlights current knowledge and critical areas for future research.

## 2. Pathophysiology of NP in SCI

NP is one of the most common and difficult complications of SCI [45]. The reported prevalence of NP in SCI ranges between 65 and 90% and frequently begins immediately following the onset of injury, with up to 75% of cases showing early NP [19,46,47,48]. Around a third of these patients regard pain as the most disabling symptom, even more so than the loss of motor and sensory function [49]. Only 4–6% of cases report improvement [50], and this pain is frequently refractory to medical treatment [16,17].

In the majority of these patients, NP occurs spontaneously and is diffuse in nature, depending on the location of the spinal injury: above, below, or at the level of the lesion [51]. Generally, NP at the level of the lesion has a more rapid onset, whereas pain above or below the level of the lesion typically has a delayed onset [46,47]. This could be because the pathophysiological mechanisms underlying the genesis of pain differ. Indeed, low-level NP appears to take longer to develop because it is associated with changes in the central nervous system, such as degeneration of spinothalamic function and decreased γ-aminobutyric acid (GABA) inhibition [48,52,53]. NP is caused by extensive molecular and plastic changes in the peripheral and central nervous systems [52,53,54,55]. These include increased neural excitability, inhibition loss [56,57,58], central sensitization [59], and glial activation [60,61,62], all of which alter pain thresholds and hypersensitivity. Additionally, cannabinoid and dopamine receptors [30,63,64,65,66,67], neurotrophic factors [68], and pro-inflammatory mediators (i.e., calcium channel) [69,70] influence the expression of NP in SCI. The pathophysiological mechanisms underlying this pain in SCI, however, remain unknown. Moving from a peripheral to central experience, the pain is associated with widespread cortical involvement of several brain areas that receive significant afferent input from nociceptive pathways (e.g., primary and secondary somatosensory cortices, prefrontal cortex, and limbic system) [71,72,73]. Neuropathy is associated with the structural and functional reorganization of the somatosensory cortex [74], the medial prefrontal cortex [75,76,77], the thalamus [78,79,80], and the anterior cingulate cortex [81,82] Additionally, the degree of somatosensory reorganization associated with altered sensory signal processing in SCI is invariably related to the intensity and duration of pain [83]. As a result, individuals who experience significant long-term structural changes in the brain are at an increased risk of developing NP. Alternatively, it is hypothesized that maintaining a low level of NP prevents the development of a neural signature of maladaptive plasticity [84,85]. Given this pathophysiological complexity, NP is exceedingly difficult to treat, and currently available pharmacological interventions are insufficient to significantly reduce NP experience during SCI [86]. As a result, individuals with SCI frequently request advancements in currently available treatments.

## 3. Pharmacological Treatment for NP in SCI

**Figure 2:**
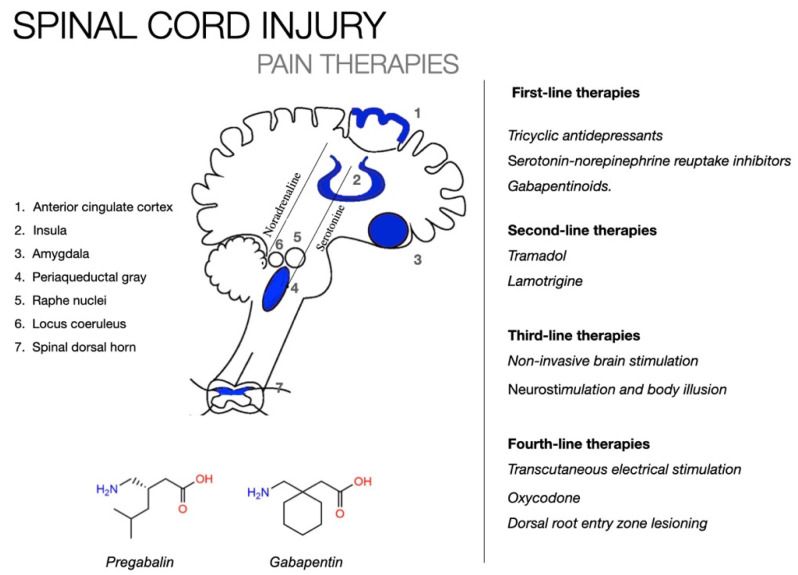
Treatment types of neuropathic pain following spinal cord injury.

This overview will not attempt to list all currently available pharmacological interventions for SCI; comprehensive reviews of pharmacological treatment are available elsewhere [49,87,88,89,90]. Instead, this work intends to provide a comprehensive overview of pharmacological treatments in order to summarize current knowledge. The objective of pharmacological treatment for neuropathic SCI pain is to eliminate or significantly reduce pain [49,87,88,89,90]. There are an increasing number of pharmacological treatments available for the condition. Each of these treatments has a unique cost–benefit profile and targets distinct pathways of pain (see Figure 2). Concerning NP in SCI, a Bayesian framework was used to summarize the primary and secondary outcomes of drug efficacy and safety [91]. Gabapentin, ketamine, BTX-A, lamotrigine, and amitriptyline had relatively high efficacy in pain relief and fewer side effects than other drugs recommended as first-line post-treatment therapy after SCI. Pregabalin and duloxetine were somewhat effective for NP and are suggested as second-line treatments, as their safety should be carefully evaluated. Tramadol, levetiracetam, carbamazepine, and cannabinoids had lower efficacy and safety profiles than the other medications, making them less suitable for post-SCI NP treatment [91].

Local anesthetics and N-methyl-d-aspartate (NMDA) antagonists are used to decrease abnormal excitability [92]. Parenteral administration of the sodium channel blocker lidocaine, in particular, has been demonstrated to be effective in the treatment of NP following SCI [93,94,95,96]. However, parenteral administration is not always feasible, and no other sodium channel blocker currently available (i.e., oral treatment) appears to be consistently effective [97,98]. Opioids, antiepileptics, and antidepressants all work by enhancing inhibitory mechanisms [99,100,101].

Opioids are one of the most widely prescribed pain medications, and they work by inhibiting pain perception by modulating both central and peripheral pain pathways [88]. Despite their superior analgesic properties, their use in NP remains contentious. Indeed, positive effects (i.e., a significant reduction in NP) [100] occur concurrently with negative effects (i.e., constipation, sedation, tolerance development, physical and psychological dependence, development of opioid-induced hyperalgesia, and the possibility of death from an overdose) and make opioid treatment not commonly recommended for patients with SCI and NP as they typically present a complex symptomatology picture [49]. Additionally, antiepileptic medications are frequently used to treat neuropathic SCI pain. Pregabalin and gabapentin are two of the most widely prescribed and approved medications on the market today and are still considered first-line treatments [91]. Pregabalin has been shown to be more effective than a placebo in patients with NP and SCI, and it is generally well tolerated [102,103]. On the other hand, it does have rare but serious side effects, as demonstrated in this case (e.g., suicidal ideation). Concerning gabapentin, previous studies conducted have revealed conflicting results [102,103]. A recent systematic review demonstrated significant improvements in NP when gabapentin was used alone in patients with SCI [104]. However, caution (i.e., dose adjustment) must be exercised in light of adverse reactions [91,105,106]. Tricyclic antidepressants are another class of drugs that are considered first-line treatments in SCI patients and have been shown to be effective in the treatment of NP [105,106]. Tricyclic antidepressants increase serotonin, norepinephrine, and weak NMDA allosteric modulators in the central nervous system, thereby modulating afferent pain signal pathways [92]. However, various adverse effects associated with the serotonergic, noradrenergic, and antihistaminic properties have been reported in this case as well [95,105]. As a result, precautions should be taken during medication dosing. The evidence for its use in patients with SCI and NP is mixed; in some cases, no effect was observed when compared to a placebo [107,108,109], while in others, it was found to be effective in alleviating at- or below-level NP, but only in a subgroup of patients with high depression scores [109]. Despite contradictory evidence, amitriptyline is considered a viable option for patients with SCI due to its relative tolerability and safety. Other antidepressants have not been studied in patients with SCI. Finally, cannabinoids have received considerable attention in recent years. Several studies have demonstrated its analgesic properties [110]. Cannabinoid receptors exert various physiological effects, including those on pain, mood, and memory [110]. While cannabinoids are generally well tolerated with mild, transient side effects, concerns persist regarding their use for NP following SCI. Rintala and colleagues found no significant difference in the relief of low-level NP when dronabinol was compared to a placebo [111]. Nonetheless, Karst et al. (2003) demonstrated significant improvements in NP using a cannabinoid analog [112]. Currently, this class of medications remains unproven as a safe and effective analgesic for NP following SCI.

## 4. A New Way: Nanomedicine

Unfortunately, despite the availability of numerous pharmacological treatments, adequate pain control is difficult to achieve, even more so in patients who have numerous other symptoms in addition to pain. The evidence indicates that medications successfully treat approximately one-third of people and only provide a 50% reduction in perceived pain [49,87,88,89,90]. As a result, current and future management must incorporate a multidisciplinary approach. Accordingly, nanomedicine has emerged as a significant area of therapeutic research. Nanomedicine seeks to improve the efficacy and safety of drugs by encapsulating them in biocompatible nanocarriers such as nanoparticles, liposomes, micelles, and dendrimers [32,113,114,115]. Nano drug delivery systems (NDDSs) can be optimized in size, shape, surface charge, and cargo dose to increase drug circulation and target specific tissues [116,117,118]. NDDSs can enhance therapeutic efficacy by regulating dosage, location, and side effects (e.g., risk of addiction) [32,113,114,115]. Indeed, nanomaterials can be used to encapsulate both free molecules and protein-based drugs, prolonging blood circulation time with controlled release, resulting in long-lasting pain relief with few side effects [37,41]. As previously stated, in conventional pain treatments, drug release was uncontrolled, requiring several uncontrolled doses daily to achieve and maintain adequate plasma concentrations. 

On the other hand, intermittent administration results in fluctuations in plasma drug concentrations, which can fall below the effective concentration or exceed the toxic concentration threshold [119]. All currently used pharmacological treatments for NP have been replicated using nanomaterials. By acting as nanocarriers for drug cargo and targeting molecules, analgesic nanoparticulate drug delivery systems can be used to alleviate NP. With regards to opioids, liposomes and polymeric nanoparticles have been used for many years to encapsulate opioids for extended release and decreased systemic toxicity [120,121,122,123], resulting in some benefits such as stabilized plasma drug levels. However, the abuse and toleration of opioids remain unchecked [124,125]. Enkephalin (ENK) is another intriguing neuropeptide analgesic [126]. ENK has been conjugated with squalene lipid to target proinflammatory mediators [126], demonstrating a greater anti-hyperalgesic effect than morphine in animal models without causing tolerance. Additionally, by using a microparticulate formulation of clustered nanoparticles, it can be delivered specifically to the brain via intranasal administration [127]. As an alternative to opioids, new pain medications targeting adrenergic, cannabinoid, and serotonin receptors are being developed [128]. For example, nanoparticles containing the synthetic cannabinoid demonstrated analgesic activity for up to 11 days following a single oral dose in a murine model of NP [129]. Additionally, NDDSs have the potential to enhance the therapeutic efficacy of local anesthetics used for perioperative pain management [116,117,118]. Local anesthetics with extended release have been developed to prolong their analgesic effect while avoiding adverse events. Numerous methods have been developed to encapsulate local anesthetics in polymeric nanoparticles (e.g., poly (lactic acid), poly (lactic-co-glycolic acid), poly (ε-caprolactone), alginate, chitosan, and copolymers), resulting in long-term stability, sustained release, and increased anesthetic efficacy in vivo [130,131,132,133]. For example, randomized controlled trials of lamotrigine for NP demonstrated efficacy in reducing pain [134,135]. Lamotrigine, on the other hand, has a poor pharmacokinetic profile due to its nonselective distribution to organs other than the brain, and its clinical applications are constrained by the risk of severe rash. NDDSs, in general, can allow for the safe use of otherwise toxic analgesic molecules [116,117,118]. However, their use may cause rare but potentially fatal systemic toxicity when they leak into the cardiovascular or central nervous systems [136,137,138]. While the majority of NDDSs are used to extend the therapeutic effect of pharmacological NP treatment, an alternative approach is to use external stimuli-responsive NDDSs to provide on-demand, personalized pain treatment [139]. This perspective may be beneficial in the management of NP associated with SCI. The location and timing of drug release can be controlled using stimuli such as light, heat, ultrasound, and magnetic and electric fields to maximize efficacy and minimize side effects. 

For example, emerging evidence suggests that using chronotherapy as a non-invasive exogenous trigger can enable precise spatiotemporal control of multiple drug administrations, thereby increasing the efficacy of nanodrugs and resulting in pain relief [132]. The term “light-activated NDDSs” refers to photosensitive molecules with labile bonds that are photochemically cleaved when exposed to ultraviolet, visible, or near-infrared light [133]. Additionally, ultrasound has been shown to be clinically useful as a non-invasive external trigger tissue penetration technique for on-demand local anesthesia [134,135,136]. As a result, regional anesthesia or peripheral nerve blocks using this technique have become the gold standard [137]. Finally, magnetic nanoparticles enabled the targeted delivery of chemotherapeutics. By controlling hyperthermia, mechanical deformation, and magnetic guiding, magnetic nanoparticles improve the spatiotemporal localization of therapeutics [129,138]. 

## 5. Nanomedicine in SCI

While it is hoped that one or more of the recommendations above will result in reliable pain relief, the reality is that many patients with SCI continue to experience NP despite ongoing treatment. This is due to a variety of reasons, one of which is related to the currently available treatment option. As briefly mentioned, nanomaterials possess unique properties that can be used to address the numerous challenges associated with NP in patients with SCI. Nanotechnology represents a significant advancement in the treatment of SCI fields [140], and current research should focus on the regeneration properties of novel materials and devices on a nanometric scale for the treatment of NP in SCI. At the moment, a number of treatments aimed at neuroprotection or axonal regeneration are being evaluated in preclinical studies, with promising results obtained when combinatorial therapies targeting two or more mechanisms of SCI pathophysiology are used [141,142,143]. In SCI models, polymeric micelles loaded with dexamethasone acetate demonstrated high efficacy in reducing the glial scar, the size of the cystic cavity in the damaged area, neuronal cell death, and promoting axon regeneration [144]. Macks et al. (2018) reported on the promising ability of polymeric micelles to transport and deliver rolipram in rats [145]. The pharmacological efficacy was increased by up to approximately 6.8 times, and it was demonstrated that it increased neuronal survival following SCI [145]. Another study reported on the use of a collagen scaffold with a microchannel pattern as a vehicle for transporting liposomes containing paclitaxel, demonstrating significant functional improvement as a result of the strong induction of neural differentiation [146]. These promising prospects for SCI repair may be a result of the multifunctional liposome’s ability to cross the blood–spinal cord barrier. Accordingly, Liu and colleagues (2010) evaluated this capacity in a rat model of SCI, indicating that liposomal transporters can overcome this barrier and concentrate near the injury site [147]. Neuroinflammation was another factor considered concerning the use of nanomedicine when treating patients with SCI. Triamcinolone acetonide has been shown to be capable of modulating neuroinflammation in the peripheral system in the in vitro models of SCI [148,149]. As indicated previously, current nanomedicine being investigated for SCI treatment aims to reduce inflammation or neurodegenerative events. However, as previously stated, nanotechnology may be able to assist patients in managing NP. Our objective with this work was to bring attention to this particular branch of therapy. We cannot say with certainty whether the path is correct and will result in the provisional or permanent resolution of NP in patients with SCI. Given the positive results of recent studies on nanodrugs in terms of NP symptoms, additional research is needed to better understand this aspect, including in conjunction with non-pharmacological treatments.

## 6. Combined Interventions with Non-Invasive Procedures

As in other fields, the brain–body disconnection after SCI has encouraged the need to integrate the advancement in nanotechnology, neurostimulation, and cell and behavioral treatments into interdisciplinary clinical treatment. Recent advances highlight the need for collaborative research at multiple levels, ranging from the molecular level to cognitive and human sciences. Substantial research has also focused on improving clinical procedures related to spinal cord regeneration to improve repair outcomes [150]. However, as most of these studies have been conducted in vitro or in vivo in animals, further investigation of the effects in humans is needed before these strategies can be used in clinical practice [150]. The persistence of NP remains one of the most devastating diseases, emphasizing the importance of initiating pharmacological and non-invasive treatments for pain relief sooner after injury [26,27,28]. A major treatment challenge is using brain–body or body–brain information. Therefore, NP may be attributed to neuroplastic reorganization. Several protocols for rehabilitation are being used in the body to reverse maladaptive plasticity and effects on the somatosensory and motor cortex after SCI [151,152,153]. Clinical treatments aimed at improving and implicitly maintaining online and offline body sensations have been shown to induce analgesic effects) [154,155]. In particular, supporting body integration processes could improve the perceptual experience and treatment of chronic pain, thereby attenuating or preventing maladaptive cortical reorganization and also reducing pain intensity [10]. The link between heterogeneous pain and disturbed body representation has been widely demonstrated, and altered afferent input appears to initiate the distortion in body representation [155].

While the exact mechanisms underlying NP following SCI remain unknown, non-invasive treatment approaches can be designed to preserve body representation and restore accurate cortical topography and corticospinal activity, preventing maladaptive plasticity and thus preventing and treating NP refractoriness.

For example, the use of bodily illusions to manipulate implicit multisensory body representations has been shown to prevent or treat secondary medical issues in people with SCI, such as inducing analgesic effects [10]. Body parts with normal or residual tactile sensations seem to be an alternative for remapping input in affected body parts. It might be beneficial to include somatotopic focal stimulation of unaffected body parts to improve embodiment and feel the body. In addition to tactile signals, other signals could also serve as mediators (interoceptive) [156,157]. Body signals may be transmitted via robotic prostheses [156,158] or virtual reality [159,160,161,162,163] in order to prevent maladaptive cortical reorganization and to alleviate pain [164]. Therefore, therapeutic approaches for any person with SCI should start in the short term after injury. If pain is thought to cause non-adaptive neuroplastic changes, techniques that comprise sensory, motor, affective, and cognitive elements may have an analgesic effect due to their ability to modify, stimulate, and regulate functional activity in multiple networks in the central nervous system.

Even non-invasive brain stimulation (NIBS) targeting specific body–brain areas can be used to alleviate chronic pain in SCI [154]. NIBS interventions have the potential to induce neural changes that counteract maladaptive plasticity at an early stage, thereby providing an important opportunity to prevent the development of more severe NP and therapeutic efficacy in pharmacology on SCI. Longitudinal studies that contribute to a better understanding of the interactions between non-invasive treatment and pharmacological interventions may contribute to a coherent and robust response to NP, with a particular emphasis on the timing of cortical reorganization following SCI. We believe that appropriate targetting of areas in the body could prevent extensive cortical reorganization, which in turn may positively affect the intensity and the chronicity of NP, permitting a better effect of nanomedicine.

## 7. Conclusions

NP following SCI frequently progresses to a chronic condition that does not respond well to a single treatment. Often, pain relief is unattainable; instead, modulation of NP may be a more realistic goal. Nanomedicine, in this sense, will become a significant area of therapeutic research. However, nanotherapeutics have only recently been investigated in the context of pain management, in part due to the complex pathophysiology of pain. Numerous diseases are associated with NP, and when they progress to chronic disease, they become difficult to treat and impose significant financial costs on the health care system. Current therapies are ineffective and have a slew of debilitating side effects. Nanomaterials and nanoparticle advancements are improving the targeting and detection of molecular sources of pain, allowing for dosage reduction while maintaining long-term efficacy and safety. Given the current treatments’ limited efficacy, combination therapy remains a critical option for preventing intractable NP. Additional research is needed to better understand this emerging field of nanomedicine and the ways in which pharmacological stimulation and body interventions can be used in combination to alter the activity of these circuits and alleviate chronic NP.

## Data Availability

Not applicable.
